# Engineering of highly potent and selective HNTX-III mutant against hNa_v_1.7 sodium channel for treatment of pain

**DOI:** 10.1016/j.jbc.2021.100326

**Published:** 2021-01-23

**Authors:** Yunxiao Zhang, Li Wang, Dezheng Peng, Qingfeng Zhang, Qiuchu Yang, Jiayan Li, Dan Li, Dongfang Tang, Minzhi Chen, Songping Liang, Yu Liu, Sheng Wang, Zhonghua Liu

**Affiliations:** 1 The National and Local Joint Engineering Laboratory of Animal Peptide Drug Development, College of Life Sciences, Hunan Normal University, Changsha, Hunan, China; 2 Key Laboratory of Hunan Province for Advanced Carbon-based Functional Materials, School of Chemistry and Chemical Engineering, Hunan Institute of Science and Technology, Yueyang, Hunan, China; 3 Key Laboratory of Molecular Biophysics of the Ministry of Education, College of Life Science and Technology, Huazhong University of Science and Technology, Wuhan, Hubei, China

**Keywords:** hNa_v_1.7, HNTX-III, peptide, analgesia, structure activity relationship, CFA, complete Freund's adjuvant, eGFP, enhanced green fluorescent protein, ICK, inhibitory cystine knot, SNI, spared nerve injury, VGSC, voltage-gated sodium channel, VSD, voltage sensor domain

## Abstract

Human voltage-gated sodium channel Na_v_1.7 (hNa_v_1.7) is involved in the generation and conduction of neuropathic and nociceptive pain signals. Compelling genetic and preclinical studies have validated that hNa_v_1.7 is a therapeutic target for the treatment of pain; however, there is a dearth of currently available compounds capable of targeting hNav1.7 with high potency and specificity. Hainantoxin-III (HNTX-III) is a 33-residue polypeptide from the venom of the spider *Ornithoctonus hainana*. It is a selective antagonist of neuronal tetrodotoxin-sensitive voltage-gated sodium channels. Here, we report the engineering of improved potency and Na_v_ selectivity of hNa_v_1.7 inhibition peptides derived from the HNTX-III scaffold. Alanine scanning mutagenesis showed key residues for HNTX-III interacting with hNa_v_1.7. Site-directed mutagenesis analysis indicated key residues on hNa_v_1.7 interacting with HNTX-III. Molecular docking was conducted to clarify the binding interface between HNTX-III and Nav1.7 and guide the molecular engineering process. Ultimately, we obtained H4 [K0G1-P18K-A21L-V] based on molecular docking of HNTX-III and hNa_v_1.7 with a 30-fold improved potency (IC_50_ 0.007 ± 0.001 μM) and >1000-fold selectivity against Na_v_1.4 and Na_v_1.5. H4 also showed robust analgesia in the acute and chronic inflammatory pain model and neuropathic pain model. Thus, our results provide further insight into peptide toxins that may prove useful in guiding the development of inhibitors with improved potency and selectivity for Na_v_ subtypes with robust analgesia.

Voltage-gated sodium channels (VGSCs or Na_v_s) are essential for the initiation and propagation of action potentials in excitable tissues such as nerve, muscle, and other excitable cells (1, 2). Structurally, all VGSCs consist of an approximately 260-kDa α subunit and associated smaller β subunits. Nine distinct VGSC α subunit subtypes (Na_v_1.1–Na_v_1.9) have been cloned from mammals and showed tissue-specific localization and functional differences. Na_v_1.1 to Na_v_1.3 are the primary VGSCs in the central nervous system, whereas the Na_v_1.6 subtype is expressed both centrally and peripherally and Na_v_1.7 to Na_v_1.9 are located in the peripheral nervous system. Na_v_1.4 is present in skeletal muscle, whereas Na_v_1.5 is primarily in the heart (3). Mutations in VGSC proteins have been associated with several diseases in human; modifying VGSC function is a useful clinical intervention strategy in states such as pain (4, 5). Pain is a pervasive and significant public health problem. There has always been a desperate need for new analgesics with higher efficacy and reduced side effects (6).

hNa_v_1.7 plays a major role in governing cellular electrical excitability as it amplifies stimuli below the threshold for action potential generation (7). In recent years, hNa_v_1.7 has emerged as a validated pain target based on clinical genetic studies, as gain-of-function mutations in the *SCN9A* gene encoding hNa_v_1.7 result in disorders of spontaneous pain and itch, including erythromelalgia, paroxysmal extreme pain disorder, and small fiber neuropathy (8, 9, 10, 11), and loss-of-function mutations of hNa_v_1.7 produce complete insensitivity to pain (12). Moreover, global hNa_v_1.7 knockout mice abolished sensitivity to thermal, mechanical, chemical, and inflammatory pain (13). Accordingly, unremitting efforts have been underway to produce analgesics with higher efficacy and better selectivity to address the large unmet medical need in chronic pain, as the existing clinical broad VGSC antagonists, such as lidocaine, carbamazepine, and phenytoin, have been reported to be effective on alleviating pain in humans and animal models but may cause side effects because of the lack of Na_v_ subtype specificity (14, 15). There exist challenges in developing subtype-selective VGSC inhibitors since VGSCs share the same domain structure with a high amino acid sequence similarity between different subtypes (16), especially attaining sufficient selectivity against Na_v_1.5 expressed in cardiac tissue and Na_v_1.4 in skeletal muscle so as not to impair normal cardiac and skeletal muscle function (17).

Spider venoms are a rich source of diverse bioactive peptides interacting with VGSCs and have attracted much attention as potential lead molecules for pharmaceutical development owing to their extremely high specificity and potency for targets (18, 19). These disulfide-rich venom peptides typically bind to the less conserved voltage-sensing domain, and hence they often achieve much better subtype selectivity than small molecules that bind to the pore region of the channel (18, 20). Efforts to develop hNav1.7 analgesics continue, including the discovery of new inhibitors (21, 22, 23, 24, 25) and modifications of the existing ones to optimize potency, selectivity, stability, and bioavailability (3, 26, 27, 28, 29). Moreover, the resolution of the spatial structure of hNa_v_1.7 greatly promoted the development of drug design and modification based on structure–activity relationship (30, 31).

Here we report the engineering of a potent and selective Hainantoxin-III (HNTX-III) analogue of hNa_v_1.7 with improved therapeutic properties in rodent models based on molecular docking. HNTX-III is a 33-residue neurotoxic polypeptide isolated from the venom of the spider *Ornithoctonus hainana* and is a member of the inhibitory cystine knot (ICK) superfamily. The ICK motif forms a rigid structure fold with unique physicochemical stability and proteolytic resistance and is an excellent scaffold for drug design (32). HNTX-III acts selectively on tetrodotoxin-sensitive VGSCs, preferentially inhibiting neuronal subtypes hNa_v_1.7 (IC_50_ 211 ± 0.022 μM) over the muscle subtype Na_v_1.4 and cardiac subtype Na_v_1.5 (IC_50_ > 10 μM) (33). Based on its potency and desirable Na_v_ subtype selectivity, we investigated the structure–activity relationship of HNTX-III and Na_v_1.7 based on molecular docking and finally engineered the H4 analogue with improved potency and selectivity against off-target Na_v_ subtypes, especially Na_v_1.4 and Na_v_1.5, as well as robust analgesic effects.

## Results

### Key activity-related residues of HNTX-III

**Figure 1:**
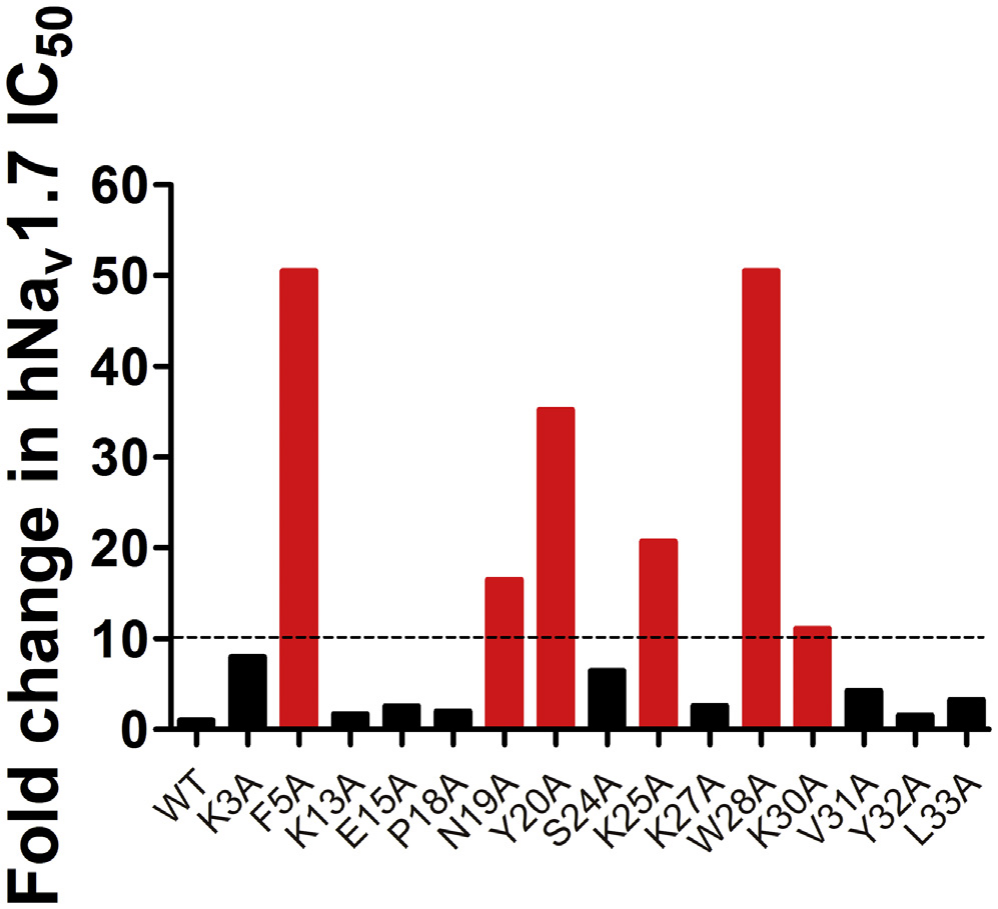
**Relative potency changes in HNTX-III Ala-scan analogues**. WT HNTX-III and mutants (excluding cysteines and native alanine) were characterized on hNa_v_1.7.

Molecular surface analysis of HNTX-III showed that the basic residues (K^3^, K^13^, K^27^, K^25^, and H^26^) are mainly distributed on one side, forming a basic patch on the surface of HNTX-III, whereas most hydrophobic residues (F^5^, P^18^, Y^20^, A^21^, V^31^, Y^32^, and L^33^) are clustered on the other side, forming a hydrophobic patch (33). To evaluate the structure–activity relationship of HNTX-III, a series of alanine substitution analogues were prepared at some amino acid residues in or near the surface of the amino acid sequence, excluding the cysteines and alanines. The inhibitory potencies of the Ala-scan analogues were determined using manual whole-cell patch-clamp recording platform on Hek293T cells transiently expressing hNa_v_1.7. Figure 1 showed that the inhibitory potencies of basic amino acid residue mutants K3A, K25A, and K30A were reduced 8-, 22-, and 12-fold, respectively. The acidic amino acid residue mutant E15A had little effect on HNTX-III inhibitory activity. Neutral amino acid residue mutants F5A, S24A, V31A, and L33A could also reduce the inhibitory activity by 135-, 7-, 5-, and 4-fold, respectively. The CD spectrum of W28A was changed compared with the native peptide, indicating that W28A reduces activity by changing the overall structure of HNTX-III (Fig. S1). Taken together, the key residues of HNTX-III binding to hNa_v_1.7 may be F^5^, N^19^, Y^20^, K^25^, and K^30^, and these mutations significantly decreased the activity; the residues K^3^ and S^24^ are also involved in the interaction, as well as V^31^ and L^33^ have moderate effect on the inhibitory activity; the residue W^28^ is necessary for the forming of the correct structure. These results indicated that the interaction between HNTX-III and hNa_v_1.7 relied on not only electrostatic force but also hydrophobic interaction force.

### The key amino acid residues of hNav1.7 interacting with HNTX-III

**Figure 2:**
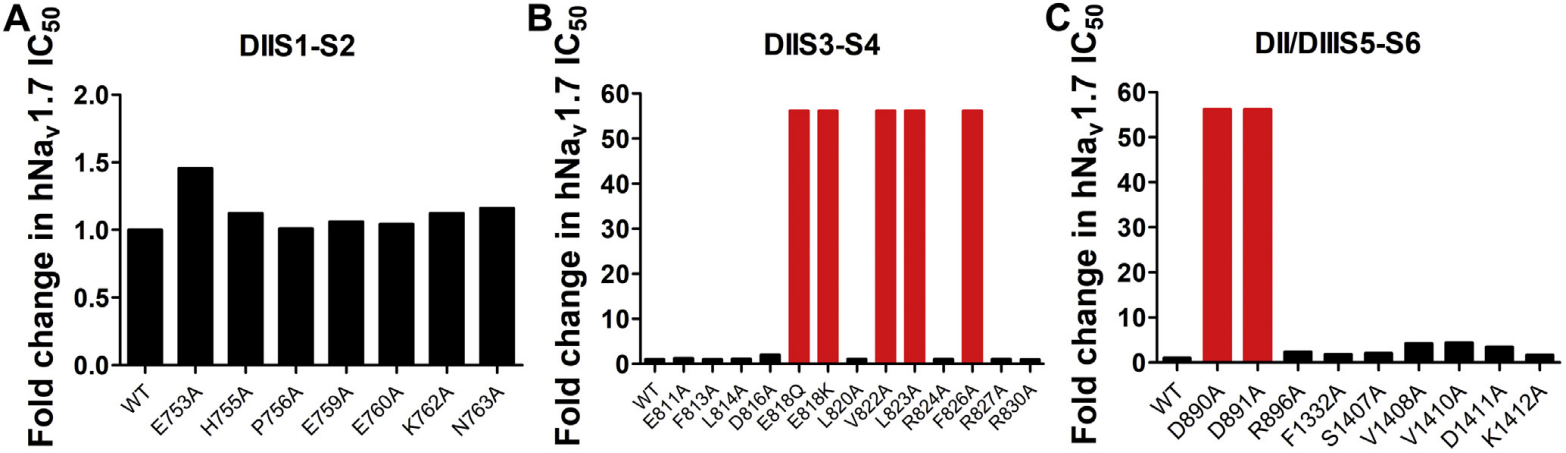
**Key residues of hNa**_**v**_**1.7 involved in interaction**. *A*, relative potency changes of WT hNa_v_1.7 and hNa_v_1.7 mutants of DIIS1-S2. *B*, relative potency changes of WT hNa_v_1.7 and hNa_v_1.7 mutants of DIIS3-S4. *C*, relative potency changes of WT hNa_v_1.7 and hNa_v_1.7 mutants of DII/DIIIS5-S6.

In our previous report, HNTX-III mainly binds to the DIIS3-S4 linker of hNa_v_1.7 (33), and the key amino acid residues on hNa_v_1.7 may play an important role in the activity or selectivity of HNTX-III binding with hNa_v_1.7. To figure out the structure–activity relationship of hNa_v_1.7 inhibition by HNTX-III, we designed a series of channel mutants to ascertain the key amino acid residues of hNa_v_1.7 binding with HNTX-III. As shown in Figure 2*A*, when the amino acid residues on DIIS1-S2 of hNa_v_1.7 was mutated to alanine, their affinity for HNTX-III did not change significantly, indicating that DIIS1-S2 does not participate in the interaction. However, mutations of E818, L822, V823, and F826 of DIIS3-S4 (Fig. 2*B*) and D890 and D891 of DIIS5-S6 on hNa_v_1.7 caused more than 100-fold lower potency, whereas V1408, V1410, and D1411 of DIIIS5-S6 caused 3- to 5-fold lower potency (Fig. 2*C*). These data suggest that the above amino acid residues in DIIS3-S4, DIIS5-S6, and DIIIS5-S6 are key sites on hNa_v_1.7 and are responsible for binding with HNTX-III.

### Molecular docking of HNTX-III/hNav1.7 VSD-II complex

**Figure 3:**
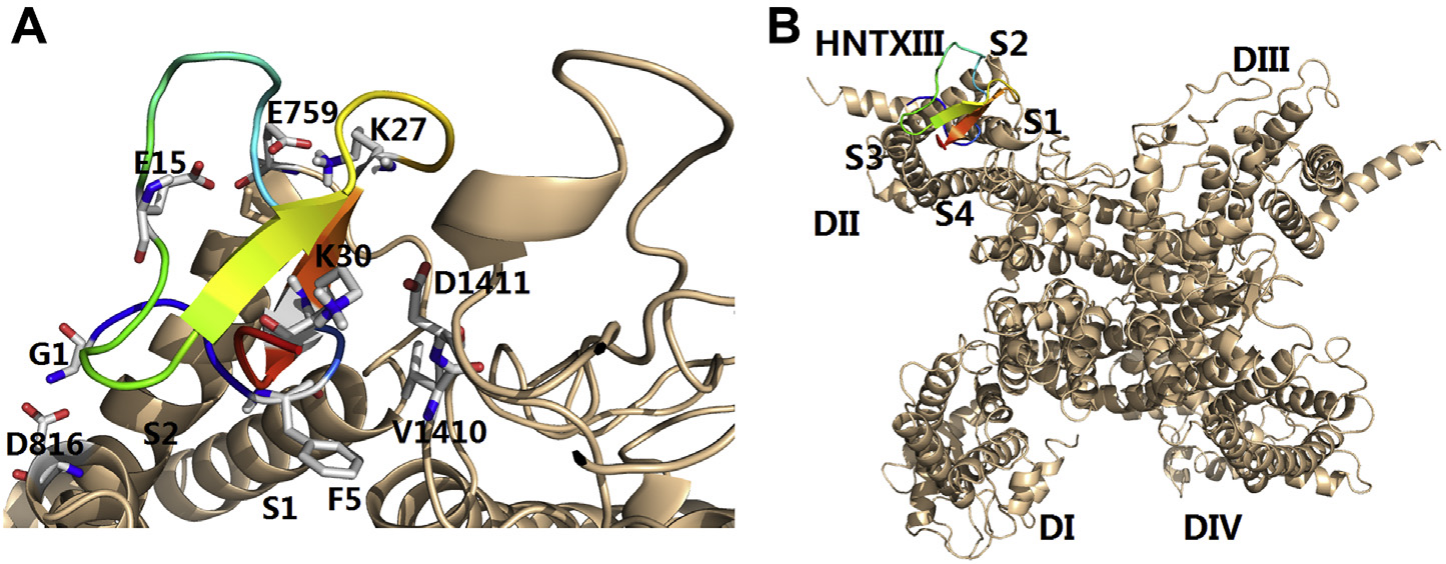
**Mode of HNTX-III action on hNa**_**v**_**1.7 VSD-II.***A*, model of the HNTX-III/VSD-II complex. *B*, position of HNTX-III relative to the full-length α subunit of Na_v_ channel.

To better understand the binding interface between HNTX-III and hNa_v_1.7 and assist molecular design, we established a molecular docking model according to the above results (Fig. 3*A* and *B*). In the docking model, the complex shows that negatively charged amino acid residues E818, D890, D891, and D1411 on hNa_v_1.7 interacted with positively charged amino acid residues K3, K25, H26, and K30 on HNTX-III by electrostatic interaction; hydrophobic amino acid residues V822, L823, F826, V1408, and V1410 on hNa_v_1.7 interacted with hydrophobic amino acid residues F5, W28, V31, Y32, and L33 on HNTX-III by hydrophobic interaction force; neutral amino acid residues N19, Y20, and S24 may form hydrogen bonds with some residues on hNa_v_1.7. These results suggest that HNTX-III is an amphiphilic molecule interacting with hNa_v_1.7 by electrostatic interaction and hydrophobic interaction.

### Substitutions of amino acid residues surrounding binding surface

According to the distribution of key amino acid residues on HNTX-III and hNa_v_1.7, key residues and a number of additional residues around the edges of interaction surface based on molecular model were selected for substitution with a variety of residues of opposite charge or varied size and shape of aliphatic and aromatic residues to explore for potential increase in Na_v_ potency. First, as shown in Table 1, the activity of mutations of the positively charged amino acid residues in HNTX-III was substantially reduced, indicating that they play an essential role in the interaction between HNTX-III and hNa_v_1.7. For example, substitution of arginine for the native lysine (K25R, 22), aspartic acid (H26D, 23), leucine (H26L, 24), and lysine (H26K, 25), respectively, for the native histidine, glutamine for the native lysine (K27Q, 26), and arginine for the native lysine (K30R, 27) resulted in analogues that show a significant decrease in affinity for hNa_v_1.7. Second, the activity of mutations of hydrophobic amino acid residues in HNTX-III also changed significantly. For example, the activity of proline at position 11 was slightly increased when it was mutated into lysine with positive charge (P11K, 10), whereas that of glutamate with negative charge (P11E, 11) was reduced by nearly fivefold; the activity of proline at position 18 mutated to leucine (P18L, 14) or tryptophan with benzene ring (P18W, 15) changed little, whereas that of lysine with positive charge (P18K, 13) increased; the activity of alanine at position 21 was increased when it was mutated to leucine (A21L, 19) or valine (A21V, 20), whereas the activity was decreased by about twofold when it was added directly after alanine (21V, 21). Last, amino acid residues near the interaction surface regulate the activity of the peptide to the channel to some extent. The activity of substitution of phenylalanine for the native glycine at position 1 (G1F, 2) or alanine (A0G1, 33), phenylalanine (F0G1, 35) added before the glycine showed little or slight decrease. However, the activity of substitution of lysine for the native glycine (G1K, 3) or lysine added before the glycine (K0G1, 34) was increased. After substitution of glycine to hydrophobic leucine (K0L1, 36) based on K0G1, the activity was improved compared with wildtype HNTX-III, but it decreased 1.7-fold compared with K0G1. These results indicate that the increase in the positive charge of the first residue and adjacent residues may enhance the activity of the peptide to the channel, while the hydrophobic interaction may destroy this binding force. In summary, K0G1 (34), P11K (10), P18K (13), N19L (17), A21L (19), A21V (20), and Y32W (29) mutants showed increased affinity of hNa_v_1.7. According to the structure–activity relationship of HNTX-III interacting with hNav1.7, the results suggest that these residues may interact with corresponding sites on target that facilitate combination.

**Table 1:** Nav inhibitory activity of HNTX-III analogs

Compound	Substitution	hNav1.7 IC_50_ (μM)	*p* Values
1	Wildtype	0.211 ± 0.049	-
2	Gly1Phe	0.282 ± 0.056	NS
3	Gly1Lys	0.150 ± 0.024	NS
4	Gly4delete	>10	<0.0001
5	Gly4Gln	0.815 ± 0.031	<0.0001
6	Phe5Trp	1.227 ± 0.167	<0.001
7	Phe5Leu	2.041 ± 0.015	<0.0001
8	Gly6Trp	0.167 ± 0.017	NS
9	Thr10Glu	1.657 ± 0.037	<0.001
10	Pro11Lys	0.127 ± 0.026	<0.01
11	Pro11Glu	0.927 ± 0.076	<0.01
12	Gly12Glu	1.891 ± 0.142	<0.0001
13	Pro18Lys	0.050 ± 0.002	<0.0001
14	Pro18Leu	0.255 ± 0.078	NS
15	Pro18Trp	0.225 ± 0.027	NS
16	Asn19Lys	0.431 ± 0.058	<0.0001
17	Asn19Leu	0.092 ± 0.046	<0.0001
18	Tyr20Trp	0.495 ± 0.039	<0.0001
19	Ala21Leu	0.133 ± 0.010	<0.01
20	Ala21Val	0.099 ± 0.019	<0.0001
21	21Val	0.512 ± 0.078	<0.0001
22	Lys25Arg	0.647 ± 0.046	<0.0001
23	His26Asp	7.58 ± 0.020	<0.0001
24	His26Leu	5.781 ± 0.117	<0.0001
25	His26Lys	>10	<0.0001
26	Lys27Gln	0.568 ± 0.042	<0.0001
27	Lys30Arg	>10	<0.0001
28	Tyr32Leu	3.076 ± 0.054	<0.0001
29	Tyr32Trp	0.075 ± 0.016	<0.0001
30	Leu33Glu	>10	<0.0001
31	Leu33Lys	>10	<0.0001
32	Leu33Phe	0.215 ± 0.029	NS
33	Ala0Gly1	0.486 ± 0.073	<0.0001
34	Lys0Gly1	0.086 ± 0.012	<0.0001
35	Phe0Gly1	0.475 ± 0.091	<0.0001
36	Lys0Leu1	0.145 ± 0.049	NS

Data are shown mean ± SD, n = 3 to 5 cells, one-way ANOVA followed by Dunnett’s post hoc tests.

NS, not significant.

### Generation of a potent inhibitor to hNa_v_1.7 by combination of single mutations

**Figure 4:**
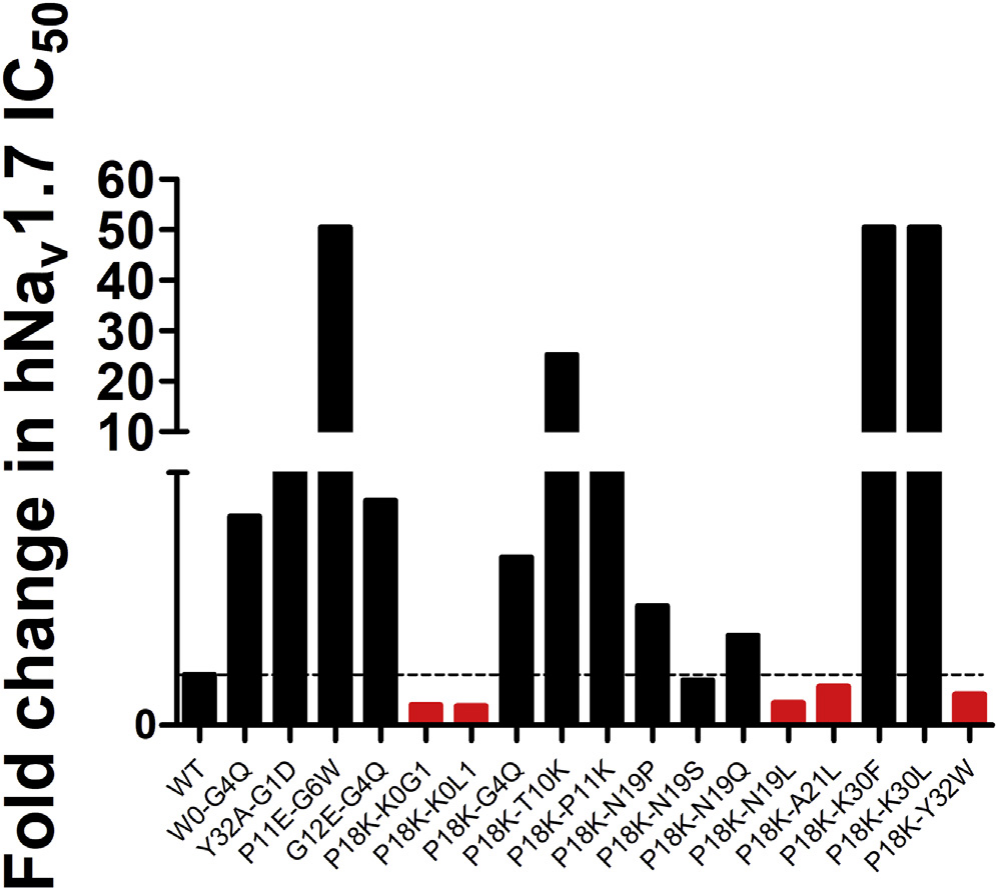
**Relative fold changes of WT HNTX-III and double mutants**. P18K-K0G1, P18K-K0L1, P18K-N19L, P18K-A21L, and P18K-Y32W showed improved affinity to hNa_v_1.7. Columns in *red* indicate that the activity of the mutant is higher than that of the wild-type HNTX-III, while columns in *black* indicate that the activity is little or significantly reduced.

Next, we performed incorporation of multiple substitutions into a single analogue based on molecular model in the hope that individual mutations would work synergistically. As shown in Figure 4, we constructed a series of double mutants mainly focused on P18K (13) in hopes of optimizing properties to hNa_v_1.7. However, the activity of most double mutants did not increase as expected, but significantly decreased, especially P11E-G6W, P18K-T10K, P18K-K30F, and P18K-K30L. Although the efficacy of P18K-K0G1, P18K-K0L1, P18K-N19L, P18K-A21L, and P18K-Y32W on hNa_v_1.7 was improved, those double mutants did not show significantly better affinity than the single substitutions. These results suggest that single mutations may contribute individually or collectively to some extent.

According to the results of mutations, we designed a series of analogues based on the molecular docking, mainly containing K0, K18, L19, L21, or V21, in hope to synergize and accumulate the antagonistic effect on hNa_v_1.7. Valine or leucine was added following A21 to strengthen the hydrophobic interaction. As shown in Table 2, the inhibitory activity of A21L-V (37) on hNa_v_1.7 was significantly increased. Although the activity of analogues with two single mutations was not significantly increased, the activity was significantly increased by the combination of multiple mutations, especially analogues containing A21L-V. Also, K0G1-P18K-A21V (38), K0G1-A21L-V (39), and P18K-A21L-V (40) showed stronger activity than single mutation. Subsequently, we designed analogues with four single mutations, namely, K0G1-N19L-A21L-V (41) and K0G1-P18K-A21L-V (42), and found that both of them significantly enhanced hNa_v_1.7 activity and that the activity of KOG1-P18K-A21L-V (42) had the largest increase (IC50 0.007 ± 0.001 μM). Then, we replaced K0G1 with K0K1 or K0L1, and A21L-V with A21V-V or A21L-L based on the amino acid sequence of KOG1-P18K-A21L-V in hope of obtaining better mutants and further obtained 43 to 48 analogues. The results showed that the inhibitory activity of 43 to 48 analogues on hNa_v_1.7 was significantly lower than that of K0G1-P18K-A21L-V, indicating that the analogues containing A21L-V were more active than those containing A21V-V or A21L-L and that K0G1 and the inserted V were more suitable for enhancing the binding of peptides to hNa_v_1.7. Finally, [K0G1-P18K-A21L-V] (named H4, 42) was found to be exceptionally potent and selective against hNa_v_1.7 with an IC_50_ value of 0.007 ± 0.001 μM. In addition, H4 had similar folding properties to wildtype HNTX-III (Fig. S2) and scale-up has been achieved for future studies (Fig. S3).

**Table 2:** Summary of potent HNTX-III analogues

ID	Peptide	Amino acid sequence	hNa_v_1.7 IC50 (μM)
1	Native HNTX-III	GCKGF GDSCT PGKNE CCPNY ACSSK HKWCK VYL	0.211 ± 0.049
37	A21L-V	GCKGF GDSCT PGKNE CCPNY **LV**CSSK HKWCK VYL	0.047 ± 0.016
38 (H0)	K0G1-P18K-A21V	**K**GCKGF GDSCT PGKNE CC**K**NY **V**CSSK HKWCK VYL	0.040 ± 0.006
39 (H1)	K0G1-A21L-V	**K**GCKGF GDSCT PGKNE CC**P**NY **LV**CSSK HKWCK VYL	0.027 ± 0.007
40 (H2)	P18K-A21L-V	GCKGF GDSCT PGKNE CC**K**NY **LV**CSSK HKWCK VYL	0.013 ± 0.002
41 (H3)	K0G1-N19L-A21L-V	**K**GCKGF GDSCT PGKNE CCP**L**Y **LV**CSSK HKWCK VYL	0.017 ± 0.005
42 (H4)	K0G1-P18K-A21L-V	**K**GCKGF GDSCT PGKNE CC**K**NY **LV**CSSK HKWCK VYL	0.007 ± 0.001
43 (H5)	K0G1-P18K-A21V-V	**K**GCKGF GDSCT PGKNE CC**K**NY **VV**CSSK HKWCK VYL	0.526 ± 0.048
44 (H6)	K0G1-P18K-A21L-L	**K**GCKGF GDSCT PGKNE CC**K**NY **LL**CSSK HKWCK VYL	0.087 ± 0.017
45 (H7)	K0G1-N19L-A21L-L	**K**GCKGF GDSCT PGKNE CCP**L**Y **LL**CSSK HKWCK VYL	0.121 ± 0.010
46 (H8)	K0K1-P18K-A21L-L	**KK**CKGF GDSCT PGKNE CC**K**LY **LL**CSSK HKWCK VYL	0.112 ± 0.027
47 (H9)	K0K1-N19L-A21L-L	**KK**CKGF GDSCT PGKNE CCP**L**Y **LL**CSSK HKWCK VYL	0.048 ± 0.022
48 (H10)	K0L1-P18K-A21L-L	**KL**CKGF GDSCT PGKNE CC**K**NY **LL**CSSK HKWCK VYL	0.022 ± 0.003

The amino acid residues in bold represent mutated residues in the amino acid sequence. Data are shown mean ± SD, n = 3 to 5 cells, one-way ANOVA followed by Dunnett’s post hoc tests.

### Molecular docking of H4/hNav1.7 VSD-II complex

**Figure 5:**
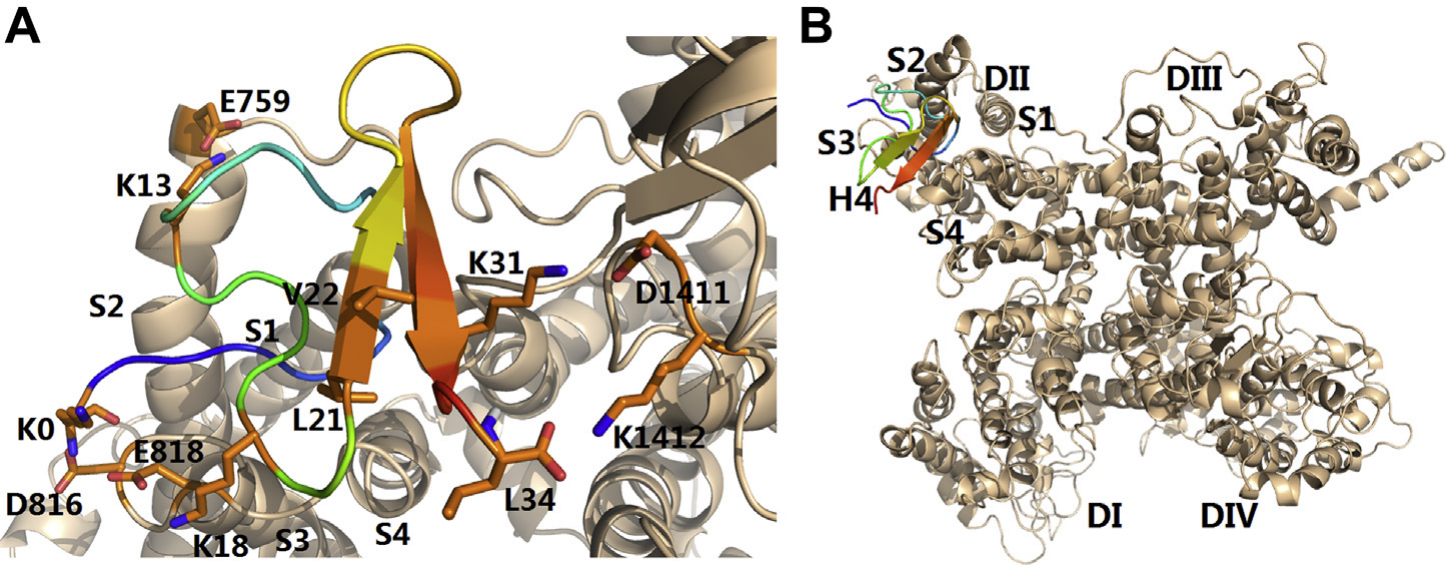
**Mode of H4 action on hNa**_**v**_**1.7 VSD-II**. *A*, model of the H4/VSD-II complex. *B*, position of H4 relative to the full-length α subunit of Na_v_ channel.

The H4 complex with VSD-II was modeled using protein-to-protein docking (Fig. 5). The complex shows that K0 and K18 make contacts with D816 and E818 by electrostatic interaction, and K13 is in contact with E759 by electrostatic interaction. In addition, L21 and V22 participate in hydrophobic interactions with V822, L823, and F826.

### Selectivity of H4 for Na_v_ and hERG (Kv11.1) channels

**Figure 6:**
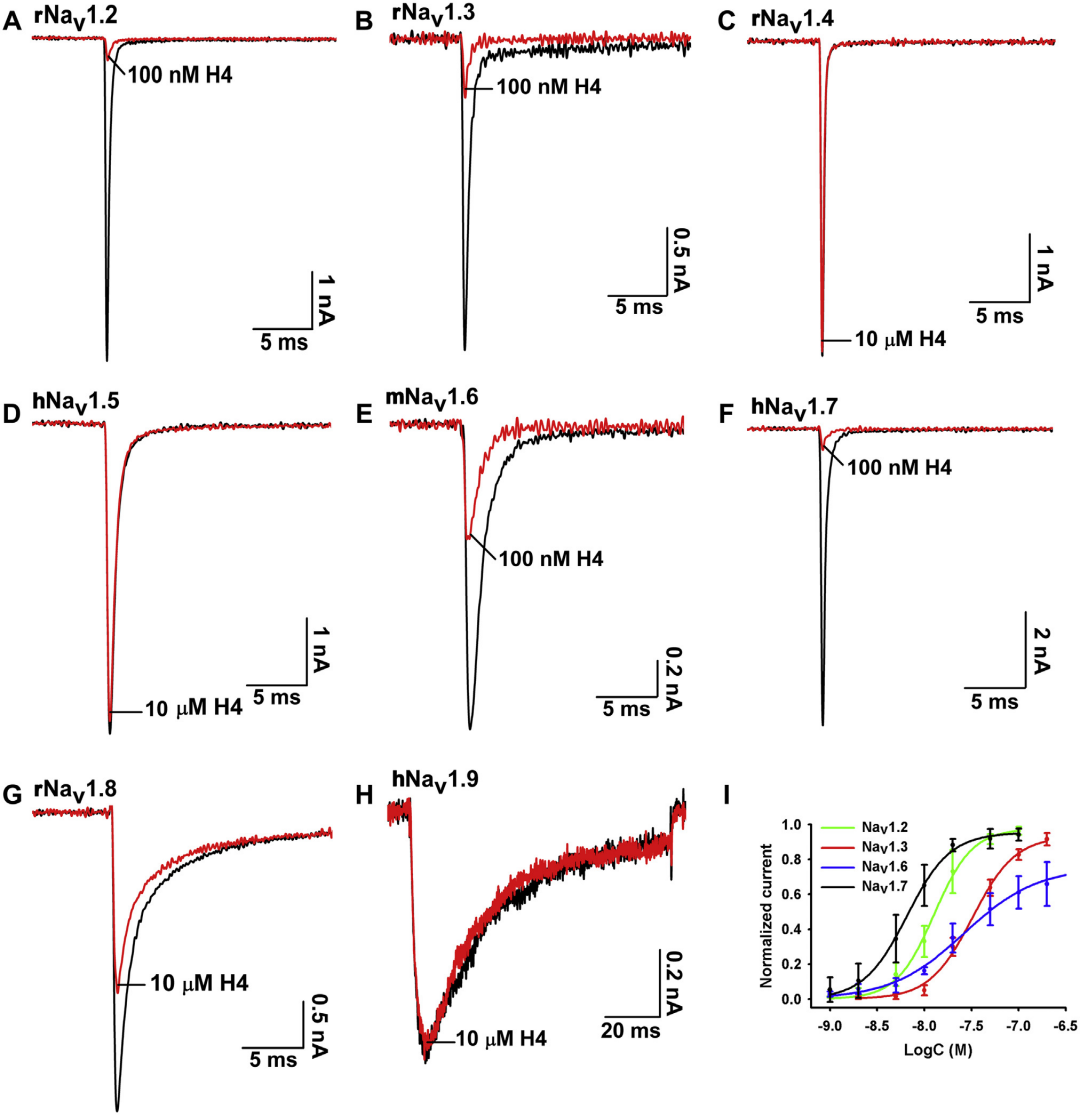
**Na**_**v**_**subtype selectivity of H4.***A*–*H*, representative current traces before (*black*) and after (*red*) addition of H4. H4 at 100 nM inhibited rNa_v_1.2, rNa_v_1.3, mNa_v_1.6, and hNa_v_1.7; 10 μM H4 modestly inhibited rNa_v_1.8 but had no effect on rNa_v_1.4, hNa_v_1.5, and hNa_v_1.9. *I*, concentration–response curves of H4 at rNa_v_1.2, rNa_v_1.3, mNa_v_1.6, and hNa_v_1.7 assessed by whole-cell patch-clamp experiments. Data are mean ± SD, with n = 3 to 6 cells per data point.

We evaluated the effect of H4 on voltage-gated ion channels by using whole-cell patch-clamp to clarify its effect on Na_v_1.2 to Na_v_1.7 channels expressed in HEK293T cells, and Na_v_1.8 and Na_v_1.9 channels expressed in ND7/23 cells (Fig. 6, *A*–*H*). H4 potently inhibited hNa_v_1.7 with an IC_50_ value of 0.007 ± 0.001 μM, exhibited two- to fivefold selectivity over other tetrodotoxin-sensitive Na_v_ channels including rNa_v_1.2 (IC_50_ 0.013 ± 0.001 μM), rNa_v_1.3 (IC_50_ 0.032 ± 0.003 μM), and mNa_v_1.6 (IC_50_ 0.023 ± 0.003 μM) (Fig. 6*I*), and exhibited over 1000-fold selectivity against rNa_v_1.4, hNa_v_1.5, and hNa_v_1.9 channels except that H4 at high concentration up to 10 μM modestly inhibited rNa_v_1.8 (IC_50_ > 10 μM). In addition, we assessed the effect of H4 on mNa_v_1.7; results showed that H4 had an inhibitory effect similar to hNa_v_1.7 with an IC_50_ value of 0.008 ± 0.001 μM (Fig. S4, *A* and *B*). We further investigated the binding kinetics of H4 on mNa_v_1.7 channel. The mNa_v_1.7 currents were slowly inhibited by H4. Of interest, H4 was also slow to wash off the mNa_v_1.7 channel, as demonstrated by a recovery of about 30% of the control current within 10 min (Fig. S5*C*). H4 at a concentration up to 10 μM had no effect on hERG (K_v_11.1) channel (Fig. S5*A*). Taken together, our data suggested that H4 was a potent inhibitor of hNa_v_1.7 channel and mNa_v_1.7 channel and showed a stable binding interaction to mNa_v_1.7 channel.

### Analgesic effect of H4 on acute pain

#### Hot plate latent pain response test in mice

**Figure 7:**
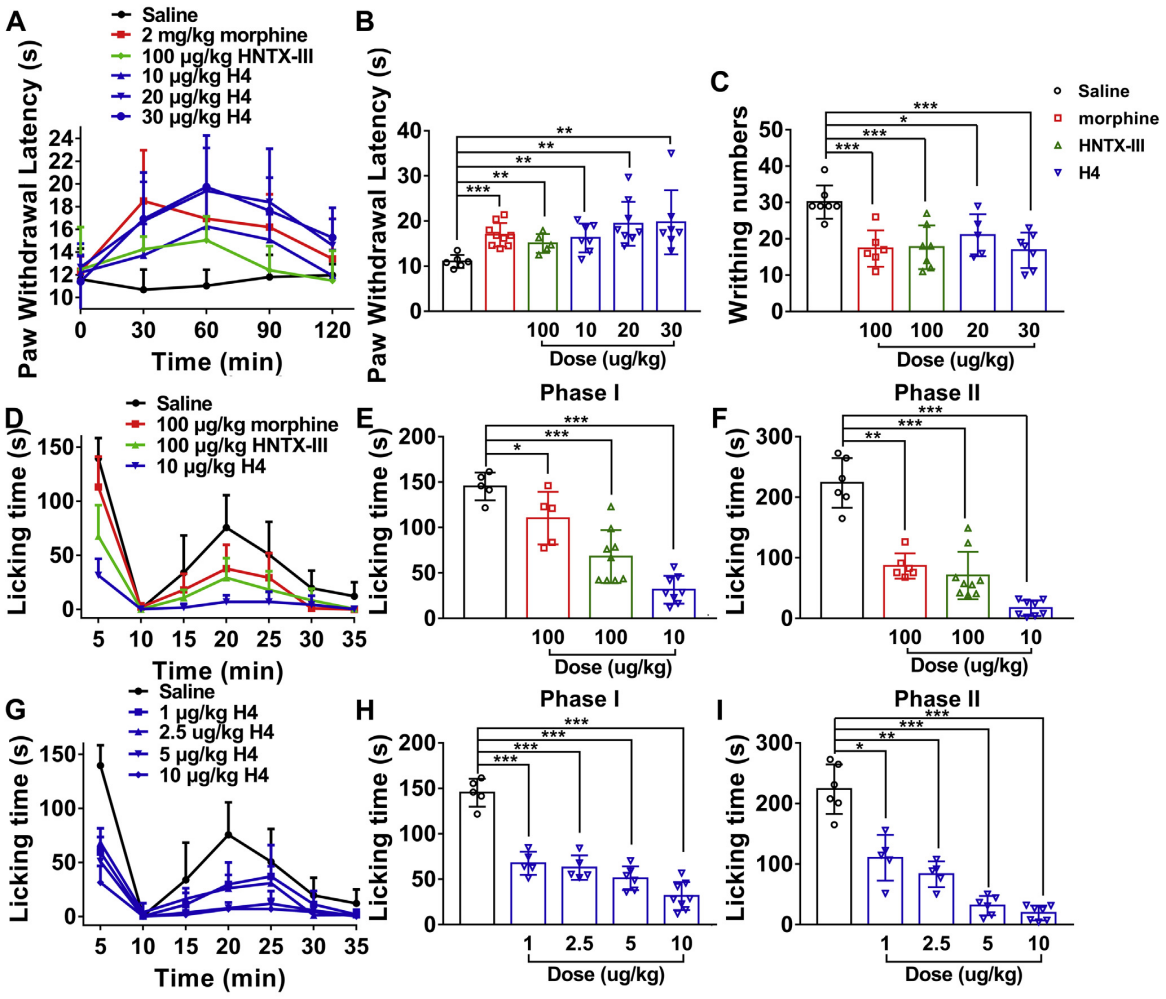
**Analgesic effect of H4 on acute pain.***A*, time course of analgesic effects of H4 (i.p.) and HNTX-III (i.p.) assessed by the hot plate test. *B*, effects of H4 (i.p.) and HNTX-III (i.p.) on acute heat responses were assessed 60 min after injection. *C*, analgesic effects of H4 (i.p.) and HNTX-III (i.p.) on acetic acid–induced writhing response in mice. *D*, time course of the analgesic effects of H4 (i.m.) and HNTX-III (i.m.) in formalin-induced spontaneous pain behaviors in mice. Evaluation of the antinociceptive effects of H4 (i.m.) and HNTX-III (i.m.) on phase I (*E*) or phase II (*F*). *G*, time course of the dose-dependent analgesic effects of H4 (i.m.) in formalin-induced spontaneous pain behaviors in mice. Evaluation of the antinociceptive effects of different doses of H4 (i.m.) on phase I (*H*) or phase II (*I*). Data are presented as mean ± SD; n = 5 to 8 per group, one-way ANOVA followed by Dunnett’s post hoc tests; ∗*p* < 0.05, ∗∗*p* < 0.01, ∗∗∗*p* < 0.001 *versus* vehicle.

In the hot plate test, a strong analgesic effect was observed after administration of H4 in a dose-dependent manner (Fig. 7*A*). Compared with the latency of control (11.1 ± 0.6 s), the latencies of mice treated by H4 (10, 20, and 30 μg/kg i.p.) for 1 h were 16.3 ± 1.2, 19.4 ± 1.6, and 19.8 ± 2.7, respectively, whereas those of HNTX-III (100 μg/kg i.p.) and morphine (2 mg/kg i.p.) were 15.1 ± 0.9 and 17.0 ± 0.9 s, respectively (Fig. 7*B*).

#### Acetic acid–induced writhing response in mice

As shown in Figure 7*C*, H4 significantly inhibits the acetic acid–induced writhing response in mice. The inhibition rates of H4 (20, 30 μg/kg i.p.) and HNTX-III (100 μg/kg i.p.) were 30.3% (*p* < 0.05), 44.1% (*p* < 0.001), and 41.2% (*p* < 0.001), respectively. As a control, morphine (100 μg/kg i.p.) caused a 42.5% (*p* < 0.001) inhibition of pain responses, which was similar to the analgesic effect of 30 μg/kg H4 and 100 μg/kg HNTX-III.

#### Formalin test

Two time phases, including the early phase (0–10 min, nociceptive pain) and the late phase (15–35 min, inflammatory pain) were recorded, respectively (Fig. 7*D*). In the control group, paw licking time was 145.2 ± 6.9 s in the early phase and 224 ±16.7 s in the late phase. In the early phase, the paw licking times were 68.1 ± 9.7 and 31.6 ± 5.4 s for HNTX-III (100 μg/kg i.m.) and H4 (10 μg/kg i.m.), respectively, whereas that of morphine (100 μg/kg i.m.) was 110.3 ± 12.9 s (Fig. 7*E*). In the late phase, the paw licking times were 71.0 ± 13.0 and 17.2 ± 4.5 s for HNTX-III (100 μg/kg i.m.) and H4 (10 μg/kg i.m.), respectively, in contrast to that of 86.7 ± 8.5 s for morphine (100 μg/kg i.m.) (Fig. 7*F*). The data indicated that HNTX-III (100 μg/kg i.m.) showed a similar analgesic effect to morphine (100 μg/kg i.m.) and that H4 (10 μg/kg i.m.) produced a robust better analgesic effect compared with morphine (100 μg/kg i.m.), especially in the late phase.

In contrast to saline, H4 resulted in a dose-dependent suppression of pain behaviors in both the time phases (Fig. 7*G*). The H4-treated groups (1, 2.5, 5, and 10 μg/kg i.m.) were 67.6 ± 5.6, 62.9 ± 6.0, 51.1 ± 5.3, and 31.6 ± 5.4 s, respectively, compared with 145.2 ± 6.9 s of saline in the early phase (Fig. 7*H*), whereas the paw licking times were 110.4 ± 15.2, 83.3 ± 8.7, 31.5 ± 6.5, and 19.3 ± 4.6 s of H4 (1, 2.5, 5, and 10 μg/kg i.m.), respectively, compared with 223.8 ± 16.7 s of saline in the late phase (Fig. 7*I*).

### Analgesic effect of H4 on chronic pain

#### Antiallodynic effect of H4 on complete Freund’s adjuvant–induced mechanical allodynia

**Figure 8:**
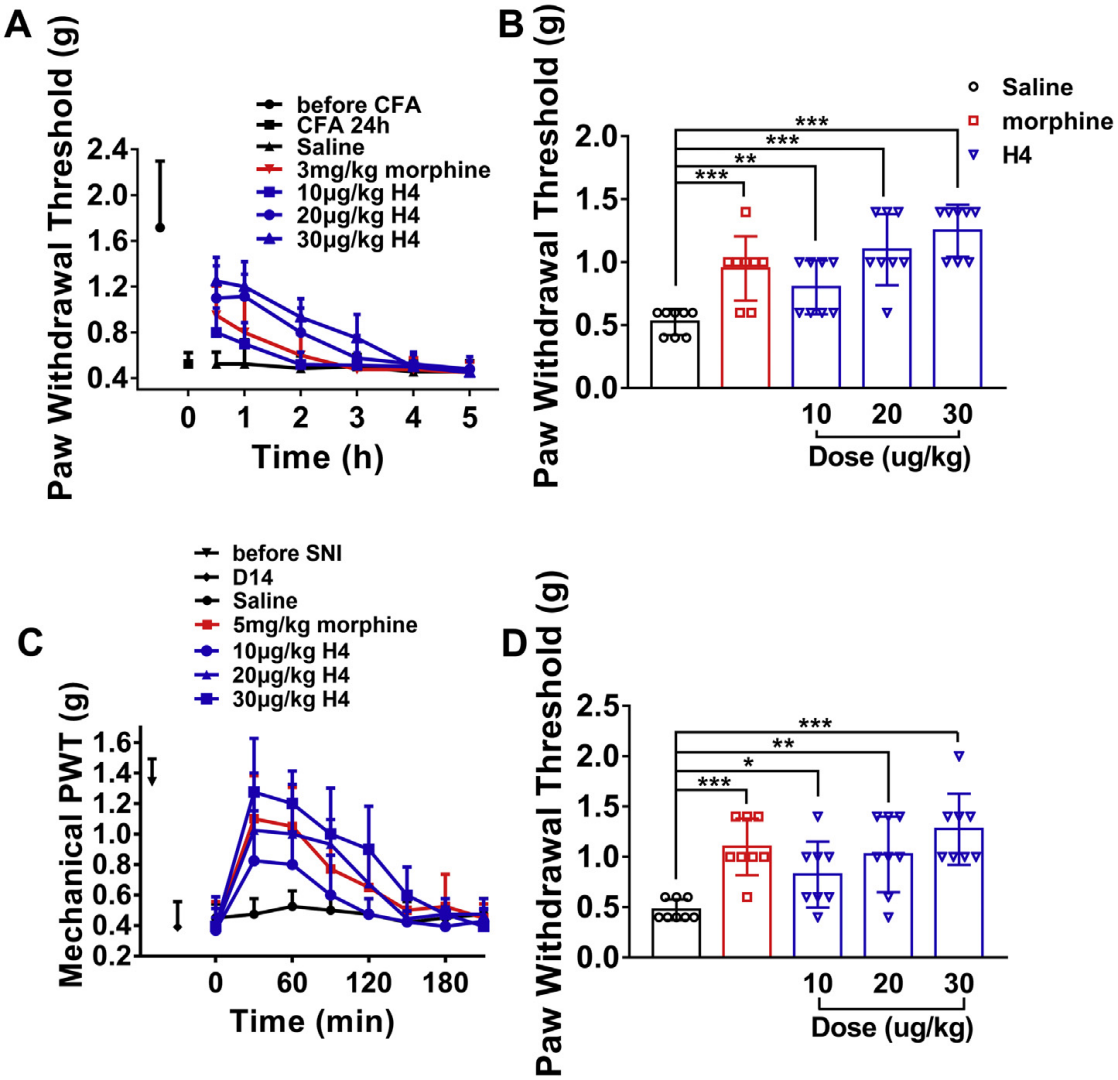
**Effect of H4 on chronic pain.***A*, time course of analgesic effects of H4 (i.m.) in the complete Freund’s adjuvant (CFA) test. *B*, evaluation of the antinociceptive effect of H4 (i.m.) assessed 30 min after the injection. *C*, time course of analgesic effects of H4 (i.p.) in the spared nerve injury (SNI)-induced allodynia model of neuropathic pain. *D*, evaluation of the antinociceptive effects of H4 (i.p.) assessed 30 min after the injection. Data are presented as mean ± SD; n = 5 to 8 per group, one-way ANOVA followed by Dunnett’s post hoc tests; ∗p < 0.05, ∗∗*p* < 0.01, ∗∗∗*p* < 0.001 *versus* vehicle.

In complete Freund’s adjuvant (CFA)-induced mechanical paw flinching and licking behaviors, H4 dose dependently reduced CFA-induced allodynia (Fig. 8*A*). H4 (10, 20, and 30 μg/kg i.m.) and morphine (3 mg/kg i.m.) at 30 min significantly enhanced the paw withdrawal threshold compared with saline (Fig. 8*B*). The anti-allodynic effects of H4 (30 μg/kg i.m.) lasted for 3 h, whereas that of morphine (3 mg/kg i.m.) lasted for 2 h. The data showed that H4 (30 μg/kg i.m.) had better analgesic activity than morphine (3 mg/kg i.m.) in inflammatory pain and that the analgesia lasts longer.

#### Antiallodynic effect of H4 on spared nerve injury model

Spared nerve injury (SNI) mice developed persistent mechanical allodynia in the ipsilateral side that appeared 3 days after surgery and remained unmodified up to 21 days (34). The effect of H4 was investigated on day 14 post surgery (Fig. 8*C*). Figure 7*L* showed the dose- and time-related antinociceptive effects of H4 and morphine on neuropathic pain induced by the mice SNI model. H4 (10 and 20 μg/kg i.p.) produced significant antiallodynic effects at 30 min, and H4 (20 μg/kg i.p.) showed analgesic effect comparable with morphine (5 mg/kg i.p.). H4 (30 μg/kg i.p.) showed a significantly stronger antiallodynic effect compared with morphine (5 mg/kg i.p.) at 30 min (Fig. 8*D*).

#### Inhibition of capsaicin-induced nociceptive behavior by H4

**Figure 9:**
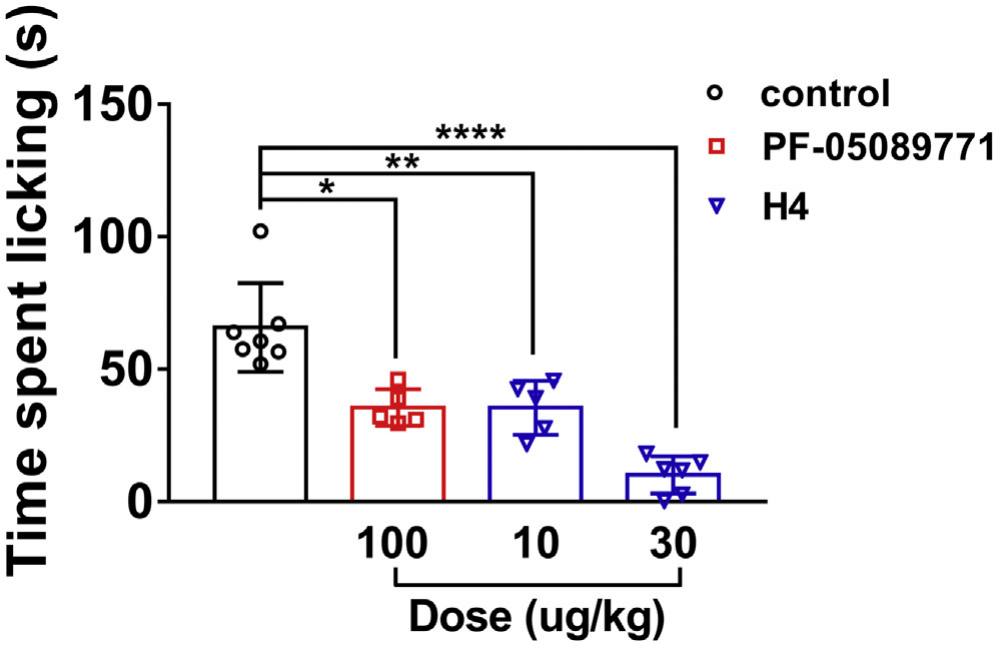
**Total time spent engaged in paw licking behavior over a 5-min test session in response to an intradermal injection of capsaicin.** Prior to capsaicin injection, mice were administered H4 (i.m.), the selective Na_v_1.7 antagonist PF-05198007 (i.m.), or a vehicle control (i.m.). ∗*p* < 0.05, ∗∗*p* < 0.01, ∗∗∗∗*p* < 0.0001 *versus* vehicle; one-way ANOVA followed by Dunnett’s post hoc tests; mean ± SD; n = 5 to 7 per group.

Intradermal application of the TRPV1 agonist capsaicin produced a robust nociceptive response in mice as evidenced by hind paw lifting and licking behavior that persisted over the 5-min observation period. The subtype-selective Na_v_1.7 preclinical tool compound PF-05089771 (mNa_v_1.7 IC_50_ 5.2 nM) was used as a positive control (35). As shown in Figure 9, PF-05089771 (100 μg/kg i.m.) significantly blocked the capsaicin-elicited response. H4 showed a dose-dependent reduction in overall nociceptive behavior and leads to a statistically significant reduction in total time engaged in paw licking at the dose of 10 or 30 μg/kg (i.m.).

#### Evaluation of RBL-2H3 cell exposure to HNTX-III analogues

Polycationic amphipathic molecules, such as neuropeptide substance *P*, neuropeptide *Y*, or the venom peptide mastoparan have been reported to cause adverse effects like β-hexosaminidase release, mast cell degranulation, and histamine release–mediated anaphylaxis *in vivo* (36). In the RBL-2H3 cell model, C48/80 as a positive drug can induce degranulation of RBL-2H3 cells and release of β-hexosaminidase. As an amphipathic peptide, the increase in affinity of H4 is due to the increase in cation and hydrophobicity, which may cause adverse effects *in vivo*. However, as shown in Table 3, compared with C48/80, there was no significant increase in β-hexosaminidase release in RBL-2H3 cells after incubation with a high concentration of HNTX-III or its analogues at 180 μg/ml. These results suggest that HNTX-III analogues may not induce adverse effects *in vivo*.

**Table 3:** The effect of HNTX-III analogues to the release rate of β-hexosaminidase of RBL-2H3 cells

ID	Analogues	Releasing rate of β-hexosaminidase (%)
-	Control	1.13 ± 0.59
1	HNTX-III	1.68 ± 0.76
39 (H1)	K0G1-A21L-V	1.63 ±0.96
40 (H2)	P18K-A21L-V	2.06 ± 0.00
41 (H3)	K0G1-N19L-A21L-V	1.16 ± 0.18
42 (H4)	K0G1-P18K-A21L-V	2.66 ± 0.87
43 (H5)	K0G1-P18K-A21V-V	2.06 ± 0.92
44 (H6)	K0G1-P18K-A21L-L	3.16 ± 0.64
45 (H7)	K0G1-N19L-A21L-L	0.90 ± 0.55
46 (H8)	K0K1-P18K-A21L-L	1.46 ± 0.96
47 (H9)	K0K1-N19L-A21L-L	1.89 ± 0.92
48 (H10)	K0L1-P18K-A21L-L	1.37 ± 0.86
-	C48/80	15.72 ± 1.12^a^

Data are shown mean ± SD, n = 3 to 6.

a *P* < 0.0001 *versus* Control, one-way ANOVA followed by Dunnett’s post hoc tests.

## Discussion

Pain, especially chronic pain, still lacks effective therapeutic drugs. Opioids remained at high odds of addiction and tolerance (37). However, currently available small molecule drugs target the highly conserved pore domain of Na_v_ channels, leading to series of side effects. Peptides usually bind to the less conserved voltage sensor domain (VSD) of Na_v_ channels and possess high potency and selectivity. New drug development and engineering and modification of available therapeutics are the hot spots and direction of current research (21, 29, 38, 39, 40, 41, 42).

In this study, Ala-scan analysis identified the key amino acid residues interacting with hNa_v_1.7 in HNTX-III as F^5^, N^19^, K^25^, W^28^, and K^30^, and these mutations significantly decreased the activity. When phenylalanine at position 5 was replaced with hydrophobic tryptophan (F5W, 6) or leucine (F5L, 7), the activity was reduced by eight to ten times; when lysine at position 25 (K25R, 22) and position 30 (K30R, 27) was replaced to arginine with the same charge, the activity was reduced by 3.5 times and more than 50 times, respectively, indicating that there were steric hindrances between the peptide and the channel and that only amino acid residues with appropriate size of side chain groups could accurately target the site; the activity of the asparagine at 19 was reduced by two times when it was replaced with positively charged lysine (N19K, 16), and increased when it was replaced with hydrophobic leucine (N19L, 17), which may be due to the enhanced local hydrophobic effect of the peptide binding to the channel, thereby improving the affinity; W28 may play a role in stabilizing the structure of the polypeptide itself, which is essential for the formation of a correct and spatial structure of the peptide. Based on the Ala-scan results and the NMR structure of HNTX-III, we speculated that HNTX-III is an amphiphilic molecule that interacts with hNa_v_1.7 by a hydrophilic surface consisting of basic amino acid residues and a hydrophobic surface consisting of hydrophobic amino acid residues.

To further clarify the binding sites of HNTX-III to hNa_v_1.7, we identified the key amino acid residues on hNa_v_1.7 by site-directed mutagenesis analysis. HNTX-III may interact with hNa_v_1.7 by multisite binding. The primary binding site may be located in the DII domain of hNa_v_1.7, and the DIII domain may be the secondary binding site, which may conform to multisite interaction mode of peptide toxin and channel (38, 40). In addition, the key amino acid residues involved in the interaction on hNa_v_1.7 were negatively charged and hydrophobic residues, which further confirmed that HNTX-III formed a rigid combination with hNa_v_1.7 through electrostatic interaction and hydrophobic interaction.

To further obtain analogues with increased hNa_v_1.7 affinity, we performed mutation analysis on amino acid residues located at and near the interaction surface based on molecular docking of HNTX-III and hNa_v_1.7. Results show that the activity of analogues of which charged residues were mutated is reduced, and the activity is significantly promoted while partial hydrophobic amino acid residues were mutated into positively charged residues or more hydrophobic residues. It indicates that the increase of positively charged residues near the interaction surface may enhance the electrostatic interaction between peptides and channel and the increase of hydrophobic residues may strengthen the hydrophobic interaction; the combination of the above two is expected to further improve the efficacy of peptides. However, the activity of analogues with two single mutations was not significantly increased, whereas the activity was significantly increased by the combination of multiple mutations, especially analogues containing A21L-V.

Molecular engineering guided by molecular docking of HNTX-III with hNav1.7 VSD-II identified H4 [K0G1-P18K-A21L-V] with high affinity on hNav1.7 (0.007 ± 0.001 μM) and >1000-fold selectivity against rNav1.4 and hNav1.5. The complex of H4 with hNav1.7 VSD-II is stabilized by salt bridges and hydrophobic/stacking interactions. Similar folding properties to wildtype HNTX-III make H4 easy to obtain. K0 and K18 in H4 analogue increased the net positive charge, whereas L21 and V enhanced the hydrophobic effect. The combination of the two may enhance the electrostatic force and hydrophobic force between the peptide and the channel, greatly enhancing the affinity and binding force.

Meanwhile, H4 showed robust analgesic effects on inflammatory and neuropathic pain, which is far beyond morphine at the doses tested in the animal pain models. At the dose of 100 μg/kg, which is 5 to 10 times the effective analgesic dose, H4 had no significant effect on motor function in the rotarod test (Fig. S4B). This indicated that H4 has no effect on motor function at the doses used in the efficacy studies. Moreover, the β-hexosaminidase release assay in RBL-2H3 cells suggests that a high concentration of H4 did not cause obvious adverse effects (Table 3). Since the extracellular region of sodium channel is highly homologous, H4 may target Na_v_1.1. Na_v_1.1, Na_v_1.2, and Na_v_1.3 are mainly distributed in the central nervous system, and Na_v_1.2 is associated with epilepsy (43), whereas Na_v_1.1 and Na_v_1.3 have been implicated in mechanical pain and chronic pain, respectively (44, 45). In addition, Na_v_1.8 and Na_v_1.9 are mainly distributed in the peripheral nervous system and have been regarded as potential pain targets. These indicate H4 targeting these channels may not cause side effects. Na_v_1.6, distributed in the central and peripheral nervous systems, has been previously reported to be related to infantile epileptic encephalopathy (46, 47, 48), and recent studies have reported that the gain-of-function mutation of Na_v_1.6 increased trigeminal ganglia neuron excitability in trigeminal neuralgia (49), whereas Na_v_1.6 knockdown ameliorated mechanical pain behavior in models of local inflammation and neuropathic pain (50, 51). The role of Na_v_1.6 in the pain pathway needs further investigation. Inhibition of Na_v_1.6 was considered to cause movement disorders and hind limb paralysis, whereas the dose of H4 used in this study did not cause significant adverse effects, indicating the effective analgesic doses of H4 used in the studies may not target Na_v_1.6. The *in vivo* target engagement of H4 in mice was evaluated in a Na_v_1.7-dependent behavioral endpoint. H4 exhibited a statistically significant reduction in the total time engaged in paw licking at the dose of 10 or 30 μg/kg (i.m.) in capsaicin-induced pain. Meanwhile, H4 has similar potency to hNa_v_1.7 (IC_50_ = 0.007 ± 0.001 μM) and mNa_v_1.7 (IC_50_ = 0.008 ± 0.001 μM), and H4 has a strong binding force to mNa_v_1.7 based on slow washout. Thus, H4 maintains high potency in the animal pain models. These indicated that H4 can target Na_v_1.7 to exert analgesic effect *in vivo*. There may exist two situations for H4 targeting Na_v_1.7 to produce analgesia: (1) The effective analgesic doses of H4 used in the studies are lower than Na_v_1.7 IC_50_, and we indicate that, compared with Na_v_1.1-Na_v_1.3, a low concentration of H4 should preferentially interact with Na_v_1.7 through higher affinity. Thus, the analgesic effect should be ascribed to preferential inhibition of Na_v_1.7; (2) Na_v_1.1, Na_v_1.3, and Na_v_1.7 as pain targets in the peripheral nervous system may participate synergistically in the analgesic activity of H4. However, owing to the difference in affinity, Na_v_1.7 is still the main target of H4, whereas Na_v_1.1 and Na_v_1.3 are partially involved in the analgesic process. However, since we have not determined the activity of H4 on mNa_v_1.1, mNa_v_1.2, and mNav1.3 channels, it is possible that mNav1.1, mNav1.2, and mNav1.3 may be more sensitive to H4 than mNa_v_1.7. In the development of a candidate HNTX-III-based analgesic drug targeting hNav1.7, the advantage is that HNTX-III conforms to a rigid ICK motif and is easy to be obtained by solid phase synthesis; the NMR structure of the hNav1.7 channel and HNTX-III has been resolved; the interaction site and mechanism of the two are clear; HNTX-III shows good analgesic effect. However, although HN3 has no obvious activity on Na_v_1.4 and Na_v_1.5, it has poor selectivity for Na_v_1.2, Na_v_1.3, and Na_v_1.6 channels, which may affect its analgesic effect.

In conclusion, this study provides insights into the modification of peptides and structure-based target drug design according to the structure–activity relationship of ion channel and peptide toxin and identifies strategies for obtaining the lead molecules of new analgesics with high efficiency and low toxicity.

## Materials and methods

### Peptide synthesis, oxidative folding, purification, and characterization

HNTX-III and its analogues were synthesized manually by using a Fmoc (N-(9-fluorenyl)methoxycarbonyl)/*tert*-butyl strategy and 1-hydroxybenzotriazole/2-(1H-benzotriazole-1-yl)-1,1,3,3-tetramethyluronium tetrafluoroborate/N-methylmorpholine coupling method (52). Peptides synthesis was accomplished on a 0.1-mmol scale. The terminal Fmoc group was removed by treatment with 1:4 piperidine/*N,N*-dimethylformamide (v/v). Cleavage from the resin with the simultaneous removal of side chain protective groups was accomplished by treatment with a cocktail (82.5% trifluoroacetic acid, 5% double distilled H_2_O, 5% phenol, 5% thioanisole, and 2.5% ethanedithiol) for 2 h at room temperature. The resin was then filtered, and the free peptide was precipitated in cold ether at 4 °C. After centrifugation and washing once with cold ether, the crude product was dissolved in ddH_2_O and lyophilized. The linear peptides were then purified by semipreparative reverse-phase HPLC (C18 column, 10 mm × 250 mm, Welch Materials Inc) with a 20-min linear acetonitrile gradient ranging from 20% to 40% at a 3-ml/min flow rate (Hanbon HPLC system, Hanbon Sci&Tech). The desired pooled fractions containing the linear reduced peptide were concentrated and lyophilized prior to folding. Reduced linear peptides were dissolved in ddH_2_O and diluted in freshly deoxygenated folding buffer consisting of 0.1 M NaCl, 0.1 M Tris-HCl, 5 mM GSH, and 0.5 mM GSSG. The crude folding mixture was readjusted to pH 8.0 by dropwise addition of NaOH. After incubating the solution at 25 °C for 24 h, the reaction was terminated by adding TFA to a final concentration of 0.1%. The desired oxidized peptide was isolated by RP-HPLC purification as described above. The molecular weights of the reduced peptides or oxidized peptides were checked by MALDI-TOF MS analysis (AB SCIEX-TOF/TOF 5800 mass spectrometer, Applied Biosystems). Peptides with >95% purity and correct (*m/z*) ratio were screened. The oxidative folding efficiency of each peptide analogue depends on the location of the mutation. The oxidative folding efficiency of each peptide analogue is similar to that of HNTX-III except that for those peptide analogues that contain A21L mutations are slightly reduced.

### Cell culture and transfection

HEK293T and ND7/23 cells were grown under standard tissue culture conditions (5% CO_2_, 37 °C) in Dulbecco’s modified Eagle’s medium supplemented with 10% fetal bovine serum. rNa_v_1.2, rNa_v_1.3, rNa_v_1.4, hNa_v_1.5, and mNa_v_1.6 channel plasmids were cotransfected with enhanced green fluorescent protein (eGFP ) into HEK293T cells, and β1- and β2-eGFP plasmids encoding β1 and β2 subunits were cotransfected with the hNa_v_1.7 or mNav1.7 channel into HEK293T cells while rNa_v_1.8 was transiently transfected into ND7/23 cells together with eGFP using Lipofectamine 2000 (Invitrogen) according to the manufacturer’s instructions. hNa_v_1.9 was transfected into ND7/23 cells according to a previous report (53). Cells with green fluorescence were selected for whole-cell patch-clamp recording at 24 h after transfection.

### Whole-cell patch-clamp electrophysiology

Whole-cell patch-clamp recordings were obtained from HEK293T cells or ND7/23 cells using an EPC 10 USB Patch Clamp Amplifier (HEKA, Elektronik). Cells were placed in a perfusion chamber in which a rapid exchange of solutions around cells could be performed. Suction pipettes with access resistance of 2.0 to 3.0 MΩ were made from borosilicate glass capillary tubes (VWR micropipettes; VWR Co) using a two-step vertical microelectrode puller (PC-10; Narishige). The standard pipet solution contained (in mM): 140 CsCl, 10 NaCl, 1 EGTA, and 10 Hepes (pH 7.4). The bath solution contained (in mM): 140 NaCl, 2 CaCl_2_, 1 MgCl_2_, 5 KCl, 20 Hepes (pH 7.4), and 10 glucose. All experiments were conducted at room temperature (20–25 °C). All chemicals were the products of Sigma Aldrich and dissolved in water. Data were acquired by PatchMaster software (HEKA, Elektronik). Data were analyzed by Igo Pro 6.10A software (WaveMetrics), Sigmaplot 10.0 software (Sigma), OriginPro 8 software (OriginLab Corporation), and Prism 5 software (GraphPad Software). HEK293T cells were held at -100 mV for all parameters examined. Macroscopic sodium currents were ﬁltered at 5 kHz and sampled at 20 kHz. To minimize voltage errors, 80% to 90% series resistance compensation was applied. Voltage-clamp recordings were acquired 5 min after establishing whole-cell configuration to allow steady-state dialysis between the cytoplasm and pipette solution. The Na_v_1.2-Na_v_1.7 channel currents were elicited by 50 ms depolarization potential to −10 mV from the holding voltage of -100 mV. The depolarization potential for Na_v_1.8 was +20 mV. Na_v_1.9 current was elicited by 50 ms depolarization potentials to −40 mV from the holding voltage of −120 mV in the presence of 1 μM TTX.

### Molecular modeling

Homology modeling of hNa_v_1.7 channel was performed with Rosetta cyclic coordinate descent and kinematic loop modeling applications with membrane-environment-specific energy function and a hNa_v_1.7 VSD-II channel structure (Protein Data Bank: 6N4R) as a template. A homology model of H4 was generated with the Rosetta Relax application, and the HNTX-III NMR structure (Protein Data Bank: 2JTB) served as a template. Docking of HNTX-III or H4 models to the hNa_v_1.7 models was performed with Rosetta Dock application with membrane-environment-specific energy function.

### Circular dichroism analysis

The secondary structure was determined by circular dichroism spectroscopy. Measurements were performed in the UV range of 195 to 260 nm at a concentration of 100 μM at 25 °C in H_2_O using a Jasco-810 spectropolarimeter. The mean residue molar ellipticities were calculated using the equation [θ] = θ/10lcMn, where θis the ellipticity in millidegrees, l is the optical path length of the cuvette in centimeters, cM is the peptide concentration in mole/l, and n is the number of residues in the peptide.

### Animals

Healthy C57BL/6 mice (weight 18–20 g) and ICR mice (weight 18–22 g) were obtained from the Experimental Animal Center of SLac-kinda, kept at 20 to 25 °C under a 12-h light/dark cycle, and fed with standard rodent chow and water *ad libitum*. All of the experimental procedures were approved by the Animal Care and Use Committee at the Hunan Province Animal Management Office. All dosing and scoring activities were conducted by experimenters who were fully blinded to treatment conditions.

### Acute heat responses

Mice preadministered H4 (10, 20, 30 μg/kg i.p.), morphine (2 mg/kg i.p.), or saline were placed on a temperature-controlled Peltier plate (model YLS-21A) set at 55 ± 0.1 °C. The latency time to jump, lift, and/or lick a hind paw was recorded according to a previous report (54).

### Acetic acid–induced writhing test

The acetic acid–induced writhing test was performed as described (55). Mice were preadministered with H4 (20, 30 μg/kg i.p.), HNTX-III (100 μg/kg i.p.), or morphine (100 μg/kg i.p.), whereas the control group received an equal volume of saline 15 min before intraperitoneal injection of 200 μl 1% (v/v) acetic acid. Mice were individually placed into open polyvinyl cages (30 cm × 40 cm × 30 cm). Abdominal writhes were defined as a wave of contraction of the abdominal musculature followed by extension of the hind limbs. The intensity of nociceptive responses was quantified by counting the total number of writhings between 0 and 30 min after the acetic acid administration. The percentage of inhibition was calculated by the equation as follows: Inhibition% = (Writhing Number_vehicle_–Writhing Number_drug_)/Writhing Number_vehicle_ × 100%.

### Formalin test

Mice received (20 μl, 10%) formalin intraplantarly under the ventral surface of the right hind paw as described (56). Mice were preadministered with H4 (1, 2.5, 5, 10 μg/kg i.m.), HNTX-III (100 μg/kg i.m.), morphine (100 μg/kg i.p.), or vehicle 30 min before injection of the formalin. Mice were observed from 0 to 5 min (phase I) and 10 to 35 min (phase II) post formalin injection. The amount of time spent licking the injected paw was recorded with a digital stopwatch.

### Complete Freund’s adjuvant–induced inflammatory pain model

Mice were restrained by hand and injected with 30 μl CFA into the intraplantar surface of the right hind paw as described (57). Mechanical paw withdrawal thresholds were measured before and 2 days after the CFA injection using manual von Frey (Stoelting Co) using the up–down method. H4 (10, 20, and 30 μg/kg i.m.), morphine (3 mg/kg i.m.), or vehicle were injected on day 2 after CFA injection.

### Spared nerve injury model

The spared nerve injury was produced as described (58). Mice (male, weighing 18–20 g) were anesthetized with sodium pentobarbital (60 mg/kg i.p.). The left hind limb was immobilized in a lateral position and slightly elevated. Incision was made at mid-thigh level using the femur as a landmark. Aseptic techniques were used, and the sciatic nerve and its three terminal branches, including the sural, common peroneal, and tibial nerves, were exposed without stretching nerve structures. Both tibial and common peroneal nerves were ligated and transacted together leaving the sural nerve intact. The common peroneal and the tibial nerves were tight-ligated with 6.0 silk, and a 1- to 2-mm section of the two nerves was removed. Great care was taken to avoid any contact with or stretching of the intact sural nerve with surgical tools. Muscle and skin were closed in two distinct layers. The sham procedure consisted of the same surgery without ligation and transection of the nerves. Intense, reproducible, and long-lasting mechanical allodynia-like behavior is measurable in the noninjured sural nerve skin territory. Mechanical allodynia was assessed by placing mice on an elevated wire mesh grid and stimulating the plantar aspect of the left hind paw with calibrated von Frey filaments (Stoelting) by using an up–down paradigm before surgery and on postoperative days. Mechanical sensitivity was determined by applying a series of the first 11 calibrated von Frey filaments (0.008–2 g) perpendicularly to the plantar aspect of the left hind paw with sufficient force to cause filament bending. Mice that developed allodynia, as defined by a significant decrease in their mechanical threshold using the von Frey filament, were used.

Mice were preadministered with H4 (10, 20, and 30 μg/kg i.p.), morphine (5 mg/kg i.p.), or vehicle, and mechanical paw withdrawal thresholds were measured at 0.5, 1, 1.5, 2, 2.5, 3, and 3.5 h post injection.

### Rotarod test

The spared nerve injury was produced as described (59). The effect on motor performance was evaluated using an accelerating rotarod (model 47700, Ugo Basile) in which normal mice were placed on a rotating drum with the speed increasing from 4 to 40 rpm over 5 min, forcing them to walk forward to avoid falling. The time (seconds) to falling was measured. Training sessions were carried out 1 and 2 days prior to the experiments, with three trials on each day. On the experimental day, a baseline response was obtained and the effects on motor performance of H4 (100 μg/kg i.p.), haloperidol (1 mg/kg i.p.), or saline administered was assessed 30 min post injection. Haloperidol is a dopamine antagonist that interferes with motor coordination and decreases rotarod performance and serves as a positive control (60).

### Capsaicin-induced nociception in mice

On the day of testing, ICR male mice were treated by intramuscular injection with equal volume of saline, H4 (10 and 30 μg/kg i.m.), or a positive control in the form of the potent Na_v_1.7 antagonist PF-05198007 (100 μg/kg i.m.) (35). Thirty minutes following test article treatment, animals were subjected to intraplantar injection of 1 μg of capsaicin in a volume of 20 μl of 5% EtOH in saline into the right hind paw. Immediately after the capsaicin injection, the total time spent engaged in licking of the injected hind paw was manually recorded over a 5-min period. All dosing and scoring activities were conducted by experimenters who were fully blinded to treatment conditions.

### β-Hexosaminidase release

β-Hexosaminidase release was performed as described (61). RBL-2H3 cells were cultured in Dulbecco’s modified Eagle’s medium containing 10% fetal bovine serum in a humidified atmosphere of 5% CO_2_ at 37 °C. RBL-2H3 cells (2 × 10^5^/cell) were washed three times with prewarmed Tyrode’s buffer and pretreated with HNTX-III analogues at 180 μg/ml for 0.5 h at 37 °C in 5% CO_2._ To determine the total amount of β-hexosaminidase released, Tyrode’s buffer containing 1% (v/v) Triton X-100 was added to three or four wells. Following stimulation, the supernatant of each well was transferred into new wells and incubated with an equal volume of substrate solution (1.29 mM p-nitrophenyl-N-acetyl-β-D-glucosaminide in 150 mM Na_2_HPO_4_, 79.6 mM citric buffer) for 1 h at 37 °C. The enzyme reaction was stopped by the addition of 0.2 M glycine buffer (pH 10.7), and the reaction product was measured at 405 nm. The percentage of degranulation was calculated using the following formula:

% β-hexosaminidase release =(absorbance supernatant−absorbance vehicle)/(absorbance Triton X-100−absorbance vehicle)×100

### Data availability

All of the data are in the article.

## Conflict of interest

The authors declare that they have no conflicts of interest with the contents of this article.

## Supporting information

Supplementary materials Synthesis, refolding and characterization of H4, effect of H4 on hERG (Kv11.1) channel, and the exercise time in the forced-swim test.
