# Supplementary material for: Engineering of highly potent and selective HNTX-III mutant against hNav1.7 sodium channel for treatment of pain
Source: J Biol Chem. 2021 Jan 23;296:100326. doi: 10.1016/j.jbc.2021.100326 (PMC7988488; doi:10.1016/j.jbc.2021.100326)
Supplement: Supplementary materials — Synthesis, refolding and characterization of H4, effect of H4 on hERG (Kv11.1) channel, and the exercise time in the forced-swim test. [file mmc1.docx]

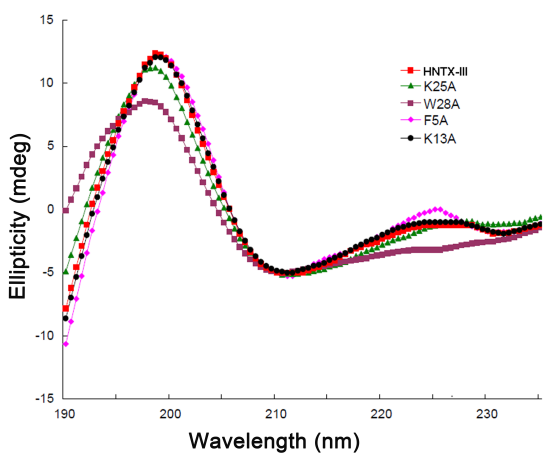


Figure S1 CD spectra of natural HNTX-III and some mutants.
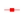
shows native HNTX-III. Except for W28A, the CD spectra of F5A, K13A, K25A and natural HNTX-III are basically coincident.


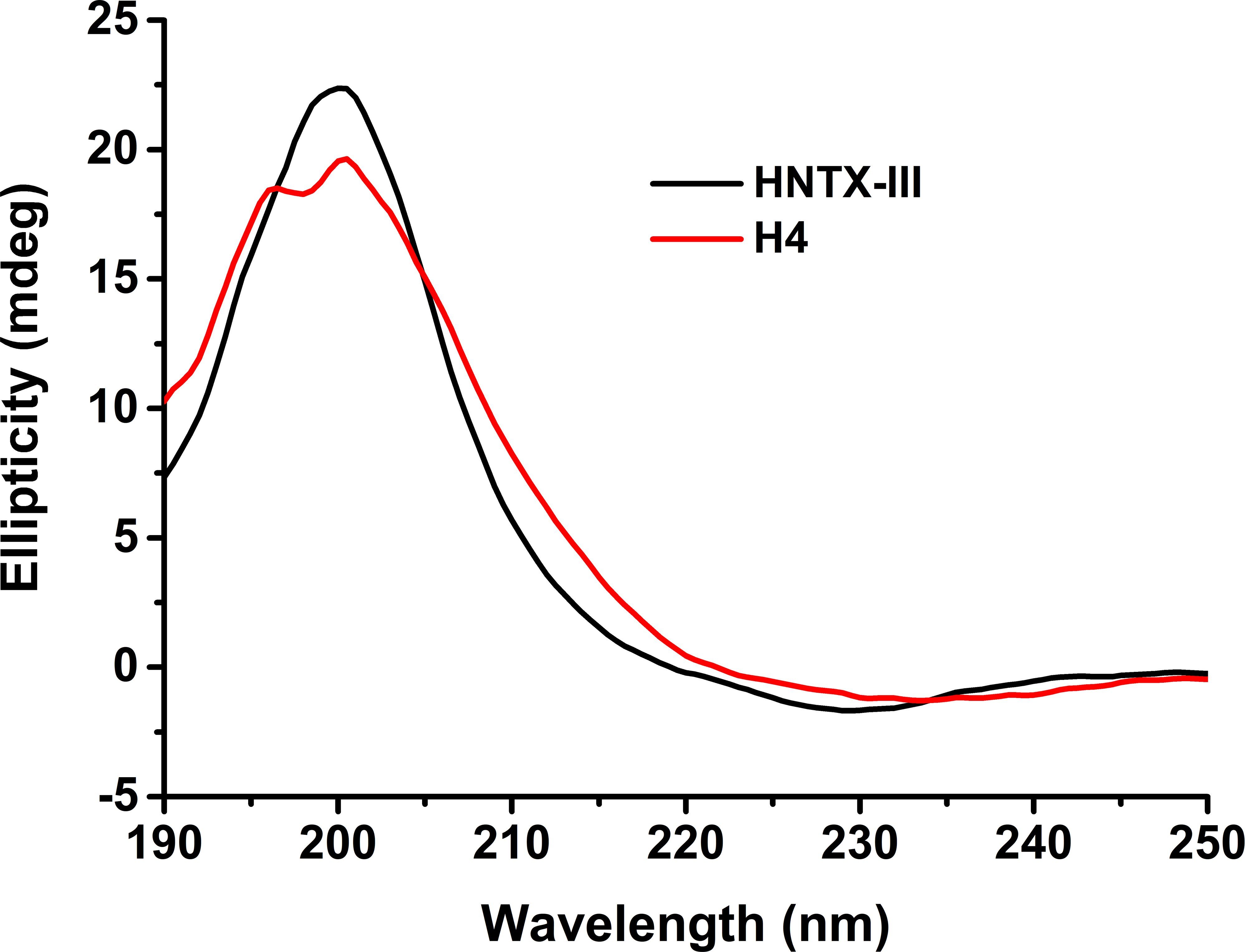


Figure S2 CD spectra of natural HNTX-III and H4.


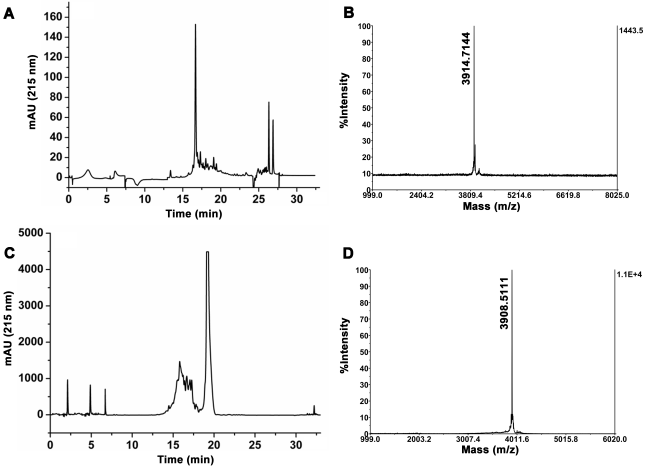


Figure S3 Synthesis, refolding and characterization of H4. (A) RP-HPLC purification of crude linear H4, and asterisked peak indicated the fraction containing linear H4. (B) MALDI-TOF MS analysis of purified linear H4. (C) Analytical RP-HPLC purification of crude folded H4, and asterisked peak indicated the fraction containing folded H4. (D) MALDI-TOF MS analysis of purified folded H4.


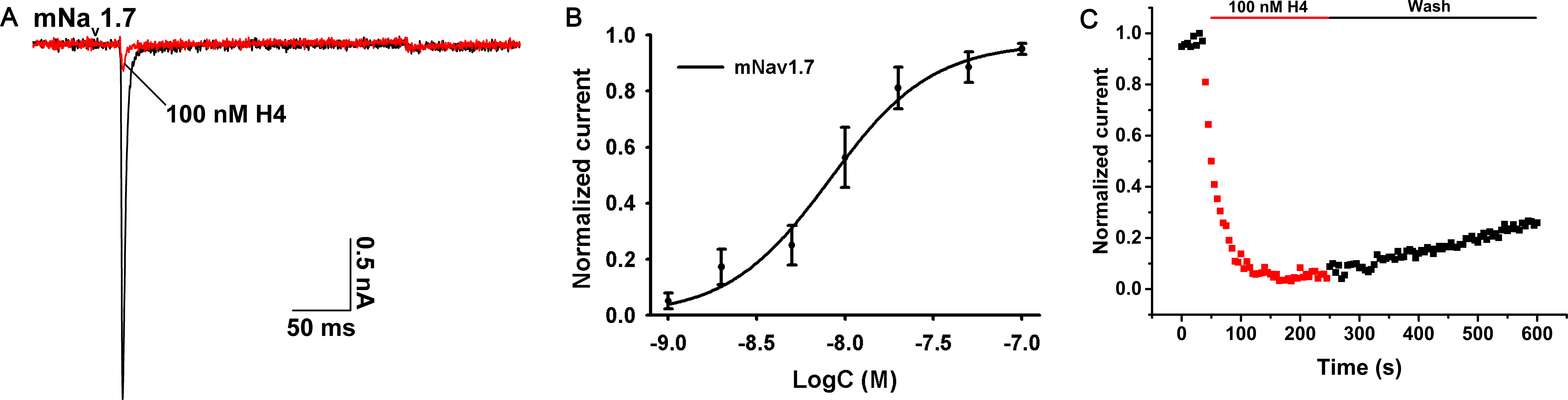


Figure S4 Effect of H4 on mNa_v_1.7 channel. (A) Representative current traces before (black) and after (red) addition of H4. (B) Concentration-response curves of H4 at mNa_v_1.7 assessed by whole-cell patch-clamp experiment. Data are mean ± SD, n=5 cells. (C) Inhibition and washout profile of mNa_v_1.7 channel by 100 nM H4 over time in the whole cell patch clamp format.


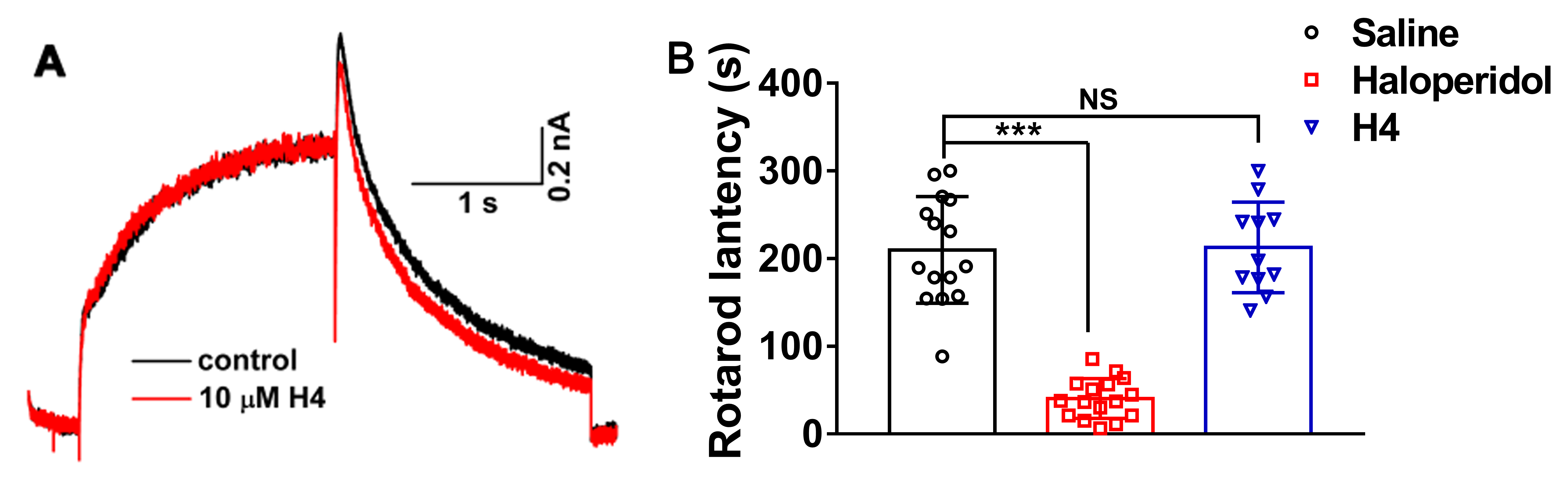


Figure S5 (A) Effect of H4 on hERG (Kv11.1) channel. 10 µM H4 showed no effect on hERG channel. (B) Effect of H4 (i.p.) on the the rotarod performance in mice (n = 11-16 per group). Data are presented as mean ± SD, one-way ANOVA followed by Dunnett’s post hoc tests; **P < 0.05, **P < 0.01, ***P < 0.001* *vs. vehicle.*
